# Development of MoSe_2_ Nano-Urchins as a Sensing Platform for a Selective Bio-Capturing of *Escherichia coli* Shiga Toxin DNA

**DOI:** 10.3390/bios8030077

**Published:** 2018-08-14

**Authors:** Jagriti Narang, Annu Mishra, Roberto Pilloton, Alekhya VV, Shikha Wadhwa, Chandra Shekhar Pundir, Manika Khanuja

**Affiliations:** 1 Amity Institute of Nanotechnology, Amity University, Noida 201313, India; annum407@gmail.com (A.M.); alekhya.v@student.amity.edu (A.V.); swadhwa@amity.edu (S.W.); 2 CNR-IC, Area della Ricercadi RM1, Via Salaria km 29.3, Monterotondo, I-00015 Rome, Italy; roberto.pilloton@cnr.it; 3 Department of Biochemistry, MD University, Rohtak 124001, India; pundircs@rediffmail.com; 4 Centre for Nanoscience and Nanotechnology, Jamia Millia Islamia University, New Delhi 110025, India

**Keywords:** fluorine doped tin oxide (FTO), molybdenum diselenide nano-urchin, geno-sensor, methylene blue (MB)

## Abstract

The present study was aimed to develop “fluorine doped” tin oxide glass electrode with a MoSe_2_ nano-urchin based electrochemical biosensor for detection of *Escherichia coli* Shiga toxin DNA. The study comprises two conductive electrodes, and the working electrodes were drop deposited using MoSe_2_ nano-urchin, and DNA sequences specific to Shiga toxin *Escherichia coli*. Morphological characterizations were performed using Fourier transforms infrared spectrophotometer; X-ray diffraction technique and scanning electron microscopy. All measurements were done using methylene blue as an electrochemical indicator. The proposed electrochemical geno-sensor showed good linear detection range of 1 fM–100 µM with a low detection limit of 1 fM where the current response increased linearly with *Escherichia coli* Shiga toxin dsDNA concentration with R^2^ = 0.99. Additionally, the real sample was spiked with the dsDNA that shows insignificant interference. The results revealed that the developed sensing platform significantly improved the sensitivity and can provide a promising platform for effective detection of biomolecules using minute samples due to its stability and sensitivity.

## 1. Introduction

The food borne infection causes serious ill effects on human health. Shiga toxin poses a serious health concern, as it causes hemolytic-uremic syndrome (HUS) which is characterized by a triad of hemolytic anemia (anemia caused by the destruction of red blood cells), acute kidney failure (uremia), and a low platelet count (thrombocytopenia) [1,2,3]. The toxin is meant to inhibit the protein synthesis, by cleaving specific base from the smaller subunit of ribosome which ultimately leads to the disintegration of ribosome assembly [4]. The detection of the toxin is vitally necessary for the prevention of severe human diseases [5]. As the toxin poses a threat to human life, the public health sector in the US began screening of processed food, such as non-intact beef for the detection of the toxin [6,7]. Conventional methods used for the detection are immune-magnetic separation and latex agglutination, and polymerase chain reactions (PCR) [8]. All of them suffer from some setbacks, such as non-specificity for immune-magnetic separation, latex agglutination, and the costly requirement of expertise and cross-reactivity in case of PCR [9]. Therefore, there is an urgent need to develop cost-effective, specific, sensitive, and accurate method for the detection of Shiga toxin. Biosensors are the best alternative technique to overcome all the setbacks associated with the conventional methods [10]. Nucleic acid based biosensors have fascinated many researchers for the employment of biological element, due to its intrinsic advantageous features [11]. DNA based biosensors offer high specificity, cost affordability, and sensitivity as compared to immune-based biosensors [12,13]. In this approach, a short stretch of DNA sequence is being immobilized onto the surface of sensing interface. Upon interaction with its complementary base pair, this produces a significant change in signal. Sensing interface used for the immobilization should have a biocompatible environment, promotes fast electron transfer, and has a large surface area for efficient immobilization [14]. Nanotechnology further enhances the advantageous features by the development of a nano-enabled interface for amplification in sensing signal [15]. Herein this approach, 2D nanomaterial i.e., molybdenum diselenide (MoSe_2_) is being employed for the development of interface. MoSe_2_ satisfies all the requirements of sensing interface as it provides amplified sensing signal, better sensitivity, and efficient immobilization. Integration of Se in molybdenum induces more conductive behavior which produces an amplified signal [16,17]. Given the above advantageous features associated with the MoSe_2_, DNA sequence-based biosensing method was proposed. Until now, there have only been a few papers on the detection of the Shiga toxin, where it has been described using electrochemical immune detection of magnetically separated Shiga pathogens. Others have described the colorimetric detection of Shiga toxin, which produces a color change when interacted with the Glycopolydiacetylene nanoparticles [18,19]. Both of the methods have shown good result, but the colorimetric detection may produce pseudo-results and magnetically separated pathogens may be non-specific. In order to provide specificity, DNA and MoSe_2_ were integrated together for the specific detection of a long stretch of target DNA having a sequence of Bacterial Shiga toxin. MoSe_2_ can absorb the nucleo-bases via van der Walls forces which made the sensing platform more compatible. In the proposed strategy, Methylene blue (MB) was used as hybridization indicator as it shows differential interaction with the ssDNA and dsDNA. The developed biosensor showed better results in terms of detection limit, repeatability and applicability in real samples.

## 2. Experimental

### 2.1. Materials

Synthesis of MoSe_2_ nano-urchin was done by using sodium molybdate (Na_2_MoO_4_·2H_2_O with 99% purity), selenium (Se with 99% purity) and hydrazine hydrate (N_2_H_4_·H_2_O) of analytical grade, purchased from Fisher Scientific. Potassium ferricyanide (K_3_[Fe(CN)_6_]), potassium ferrocyanide (K_4_[Fe(CN)_6_]), potassium chloride (KCl) and Methylene blue (MB) of analytical grade were purchased from Fisher Scientific, Noida, India. The synthetic oligonucleotides of 18-mer was purchased from GCC Biotech (India Pvt Ltd., West Bengal, India) with their base sequences:

- Capture probe—5′-NH_2_-AAACTCAAAGGAATTGAC-3′;
- Target probe—3′-TTTGAGTTTCCTTAACTG-5′.

The oligonucleotides solution of different concentrations was prepared in TE buffer (Tris-EDTA Buffer—10 mM Tris, pH 8.0, 1 mM EDTA). The probe DNA sequence of *Shiga pathogen* used in the present study is of Shigella species, and the whole genomic sequence can be found in Genbank (https://www.ncbi.nlm.nih.gov/pmc/articles/PMC127526/) [20].

### 2.2. Apparatus and Methods

Differential pulse voltammetry (DPV) measurements were performed on a Potentiostat/Galvanostat (Autolab, Eco Chemie, Dutch, The Netherlands. Model: AUT83785) and driven by NOVA 1.8 software [21]. Fluorine doped tin oxide (FTO) electrode was used as the working electrode, Pt as a counter electrode and Ag/AgCl as a reference electrode, entire as a three-electrode system for the electrochemical measurements at different parameters (Modulation amplitude: 0.02500 V; Modulation time: 0.05000 s; interval time: 0.50000 s; step potential: 0.00500 V and scan rate: 0.01000 V/s) throughout the experiment. For the morphology of nanostructures, Scanning Electron Microscopy (SEM Zeiss, Sigma, St. Louis, MO, USA) and Fourier-transform infrared (FTIR) spectra in a Shimadzu 8700 FTIR spectrophotometer analysis was done to identify organic, polymeric, and in some cases, inorganic materials.

In order to see the MoSe_2_ peak differences corresponding to the number of layers present in the sample, RAMAN Spectroscopy was performed. Cyclic voltammetry (CV) and differential pulse voltammetry (DPV) was performed for the electro analytical measurements on a Potentiostat/Galvanostat (PGSTAT204).

### 2.3. Synthesis of 2D Nanomaterial MoSe_2_ Nano-Urchins

Synthesis process for MoSe_2_ of pH 8.86. Initially, 0.02 mole of sodium molybdate (Na_2_MoO_4_) is dispersed with 50 mL of deionized water (DI) at room temperature (RT) and magnetically stirred for 10 min to give a clear solution. In separate beaker 0.04 mole of Se powder is mixed with 10 mL of hydrazine hydrate (N_2_H_4_·H_2_O) at RT and stirred magnetically for 15 min to give a black suspension. This black-colored suspension is slowly dropwise added to the clear colorless solution of sodium molybdate and DI at RT stir violently for 20 min, an orange-brown solution is obtained. The pH of the solution thus obtained should be nearly 10. Use 2 mL of acetic acid to change the basicity of the solution. The pH thus obtained should be 8.86 pH. Afterward, transfer the solution to Teflon lined autoclave, place in hydrothermal at 200 °C for 24 h. After completion of 24 h, the autoclave is allowed to cool to room temperature [22]. Then, the solution is washed with DI water followed by acetone and then dry vacated at 40 °C for 120 min. The sample is ready in powder form for characterization.

### 2.4. Preparation of Electrode

The FTO electrodes were diced having sizes 1 cm × 2 cm using a diamond cutter [23]. The bare FTO electrode was cleaned gently with a mild detergent solution. Subsequently, it was washed several times with distilled water and ethanol followed by air dry at room temperature. The surface conductivity was measured using multimeter. Afterward, the prepared MoSe_2_ were drop deposited evenly onto the surface and was kept for air-drying.

### 2.5. Immobilization of “Amino Modified” Probe DNA onto MoSe_2_ Modified FTO Electrode

**Scheme 1:**
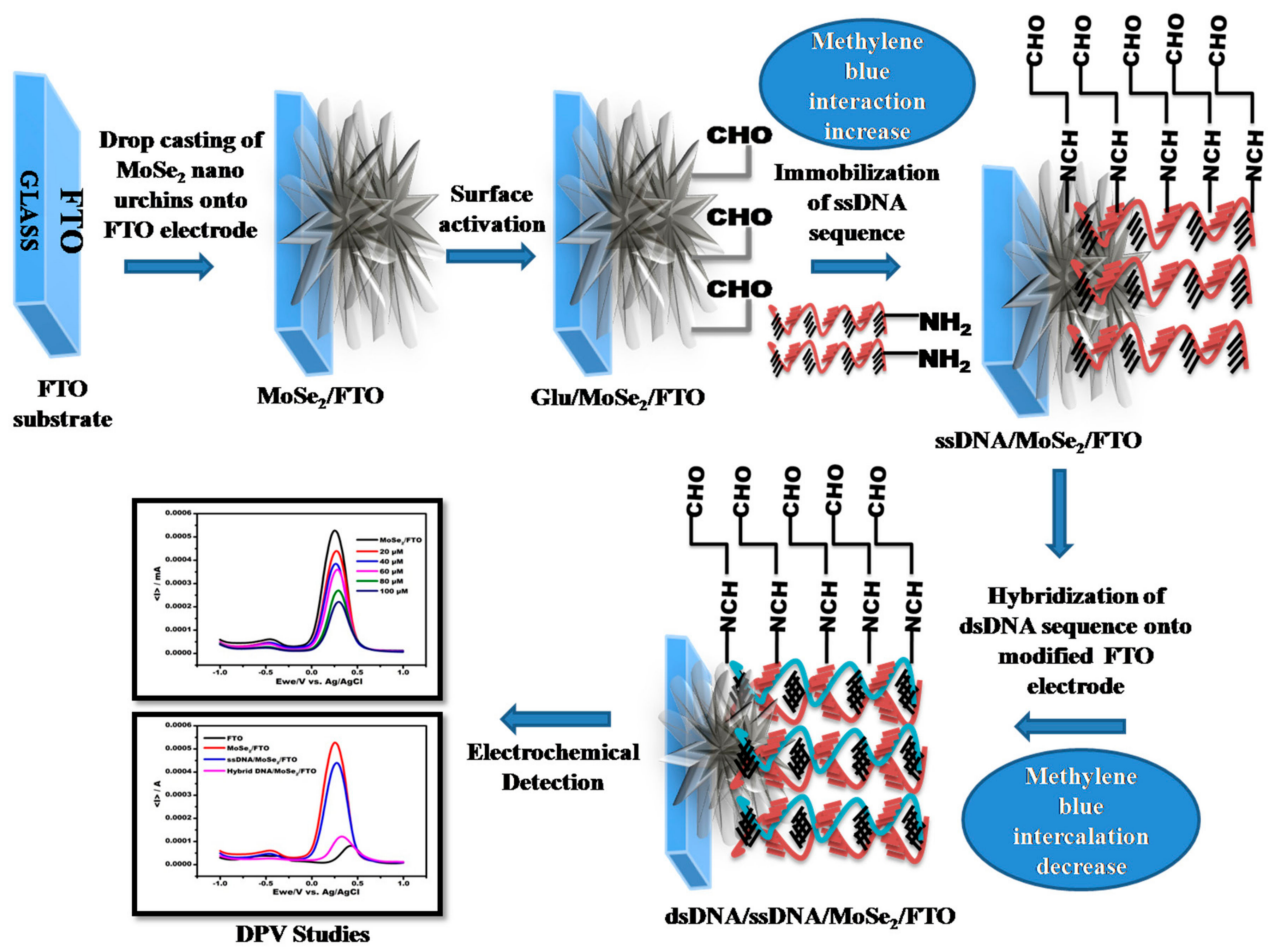
Schematic representation of DNA based electrochemical detection of *Escherichia coli* Shiga toxin using 2D nanomaterial.

MoSe_2_ modified FTO electrode was exposed to 1 mL glutaraldehyde solution (2.5%) for 12 h at RT to form Schiff base linkage followed by addition of different Shiga toxin ssDNA sequence concentrations ranging from 20–100 µΜ onto different MoSe_2_ modified FTO electrodes of same dimensions and conductivity at room temperature at 36 °C for 2 h. The electrode with an optimum concentration of Shiga toxin ssDNA sequence was used for the subsequent experiment. The -CHO group of glutaraldehyde formed linkage with -NH_2_ group modified DNA sequence. NH_2_-modified probe facilitates the “cross linking” with the bi-linker which covalently bind to amines, therefore establishing a strong link resulted in more stable attachment of biomolecule. The general method is summarized in Scheme 1.

### 2.6. Electrochemical Characterizations of Modified Electrode 

Electrochemical characterization of ssDNA/MoSe_2_/FTO electrode was done by using electrochemical setup consisting of working electrode (ssDNA/MoSe_2_/FTO), reference electrode (Ag/AgCl) and counter electrode (Pt wire) by sweeping the potential range from −1.0 to +1.0 V in 1 µM MB (1 mL) solution in 0.1 M KCl. Various parameters such as probe concentration, scan rate, response time, pH and temperature were optimized in order to obtain maximum sensing signal. Various concentrations ranging from 100 fM to 100 μM of dsDNA sequences were prepared [23]. The specificity of the proposed DNA sensor was evaluated by monitoring its response with non-complementary target DNA (salmonella dsDNA) for hybridization reactions of the probe which were used instead of target DNA sequence as same as the above-explained protocol for fabrication of modified electrode (Shiga ssDNA/MoSe_2_/FTO).

“Non Comp” DNA Sequence—3′CTACTCATAACTACGGCT5′.

### 2.7. Application of Fabricated Biosensor in a Real Sample

To validate the performance of the biosensor in real sample, fruit juice (5 mL) was treated with PBS (1 mL), pH 7.5 followed by centrifugation for 10 min at 10,000 rpm to remove debris. The obtained pellet was re-suspended in deionized water (DW). The resuspended pellet was spiked with DNA (1 fM) and exposed to probe modified FTO electrode [24].

## 3. Results and Discussions

### 3.1. Physical Characterization of MoSe_2_ Nano Urchins

**Figure 1:**
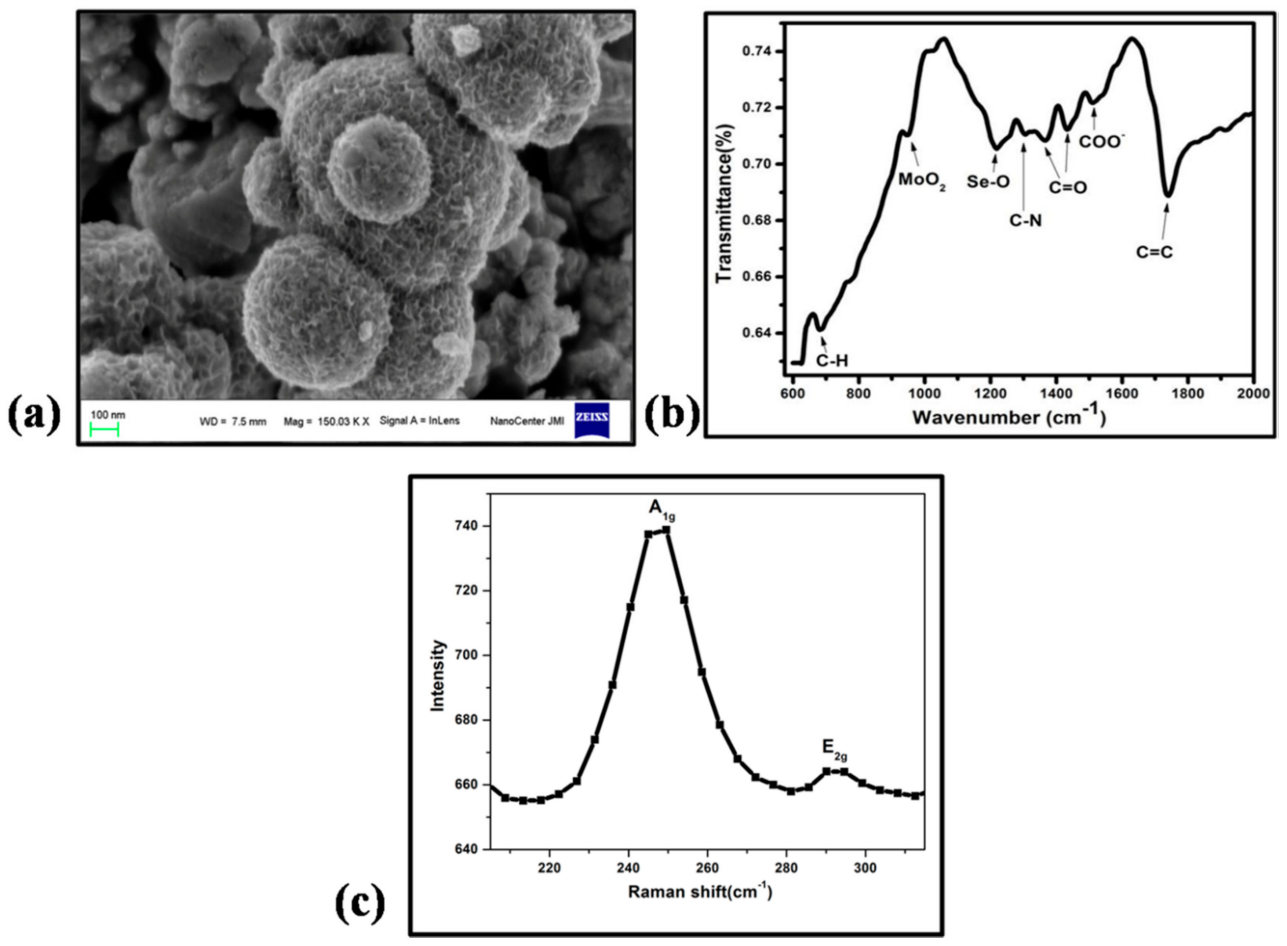
MoSe_2_ Nanourchin (**a**) Scanning electron micrograph at 100 nm focusing urchin like morphology (**b**) FTIR spectrum (**c**) Raman spectrum.

Figure 1a shows the SEM image of the MoSe_2_ sample. As evident, sample consists of homogeneously distributed microsphere of length ~10 µm and diameter ~150 nm. Microspheres arranged in a way that at one end they are closely packed and other is spread out resulting in the formation of ‘spherical’ urchin shaped morphology called ‘nanourchins’. FTIR in Figure 1b shows different band stretching and bending of the compound. The small peak is observed for Molybdenum dioxide (MoO_2_) at 947 cm^−1^. At 1218.71 cm^−1^, Se-O peak is observed. A sharp peak of C=C is observed at 1739.98 cm^−1^. Few small peaks of C-H, C-N, COO- are observed corresponding to 681.42 cm^−1^, 1305.37 cm^−1^, 1510.09 cm^−1^, respectively. Two small peaks were observed for C=O at 1365.84 cm^−1^ and 1433.36 cm^−1^, respectively. Figure 1c depicts the two Raman active modes A_1_g and E_2_g^1^ for MoSe_2_ are visible in Raman spectra. The A_1_g and E_2_g^1^ wave number are observed at 254.13 cm^−1^ & 289.75 cm^−1^. The plane A_1_g mode corresponds to the out of plane vibrations of Se atoms while the E_2_g^1^ mode corresponds to the in plane vibrations of Mo and Se. In earlier studies, it is reported that, with changing the number of layers of MoSe_2_, the A_1_g band undergoes blue shift while the E_2_g^1^ band undergoes red shift with increasing number of layers. A very sharp peak of E_2_g^1^ band for MoSe_2_ is observed that signifies the sample purity.

### 3.2. Electrochemical Characterizations of Various Stages of Electrode

**Figure 2:**
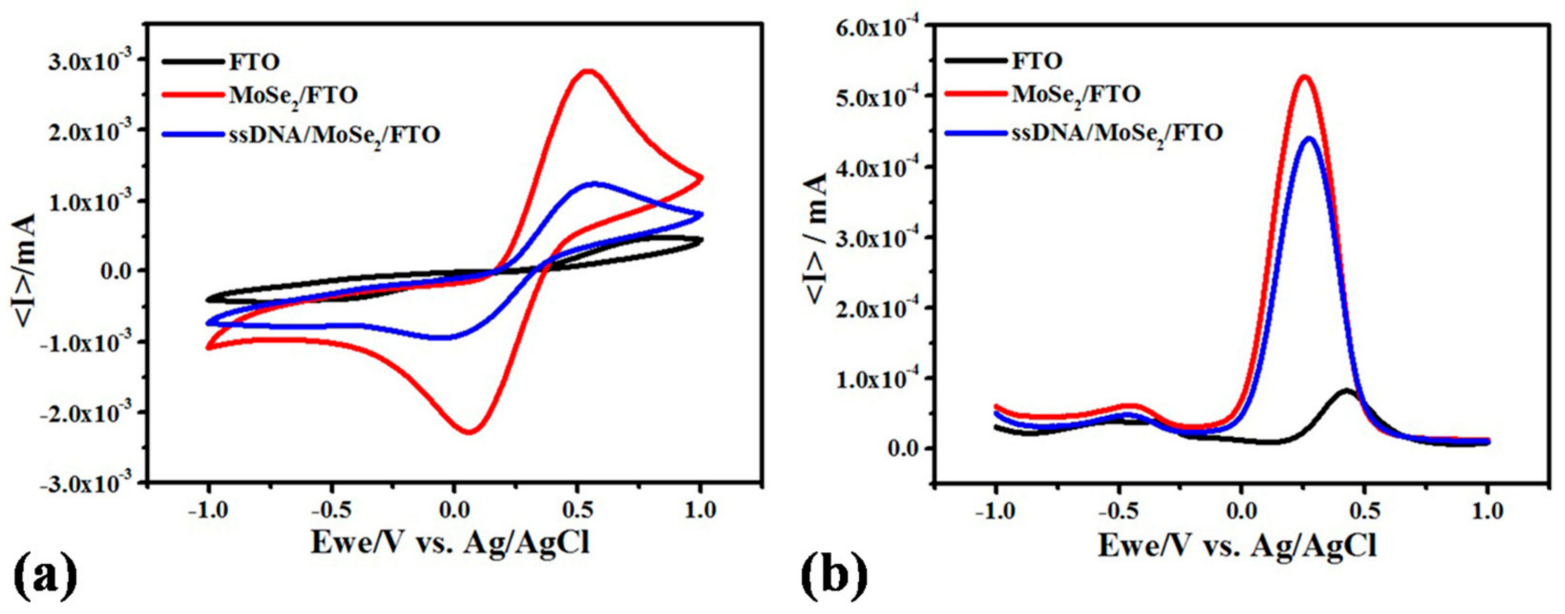
(**a**) Cyclic Voltammogram and (**b**) Differential Pulse Voltammogram of fluorine doped tin oxide (FTO), MoSe_2_/FTO and ssDNA/MoSe_2_/FTO conducted in 1 µM MB (pH 7.4) in 0.1 M KCl at the scan rate of 100 mV/s.

Electrochemical study of different stages of the electrode was conducted in 1 µM MB containing 0.1 M KCl. As depicted in Figure 2a, a well-defined anodic peak was observed in case of bare FTO electrode. A drastic increase in anodic current was observed after introduction of MoSe_2_ onto the surface of FTO which indicates the intrinsic conductive behavior of selenium in MoSe_2_. After the capturing of NH_2_ modified probe DNA onto the surface of FTO, the peak current was decreased which happens due to the blockage of electron transfer due to the insulating layer of ssDNA. The anodic current was further decreased after the addition of target ssDNA in the solution indicating that the oligonucleotides of the target DNA were successfully hybridized with the capture probe [25]. These results confirmed the successful development of different phases of the electrode.

### 3.3. Optimization of Experimental Variables

**Figure 3:**
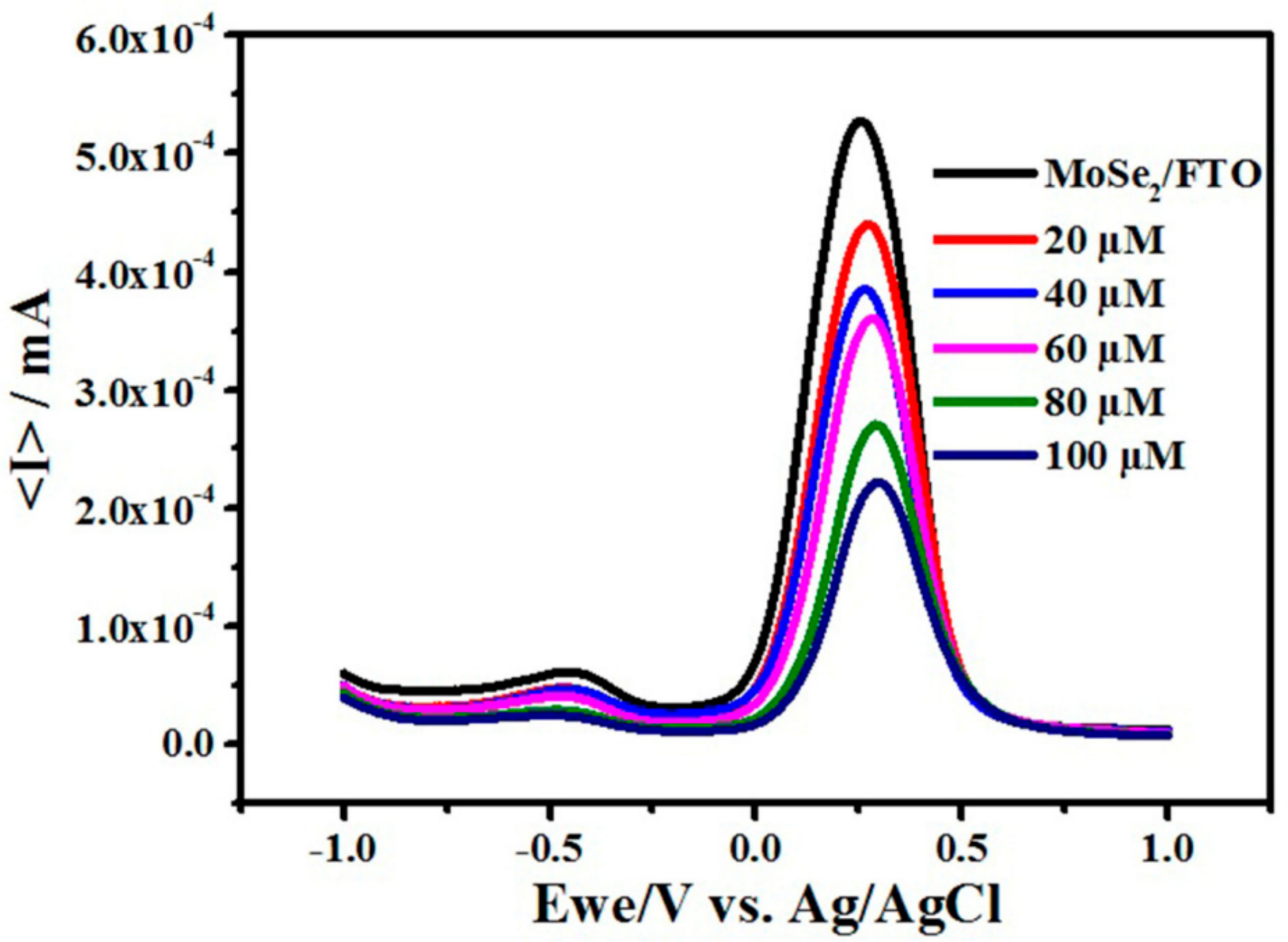
Differential pulse voltammogram of modified electrode at various concentrations ranging from 20 µM–100 µM conducted in 1 µM MB (pH 7.4) in 0.1 M KCl at the scan rate of 100 mV/s.

Various analytical parameters were optimized such as DNA probe concentration, temperature and response time for obtaining best results. DNA probe concentrations varying from 20 to 100 μM were taken for checking differential effects on the sensing signal. Figure 3 depicts that there is a decrease in oxidation of MB upon an increase in DNA probe concentration which is due to the non-conductive behavior of the biological element. For further experimental studies, it was concluded that the 40 μM DNA probe concentration was sufficient for both increased current signal and target DNA hybridization.

**Figure 4:**
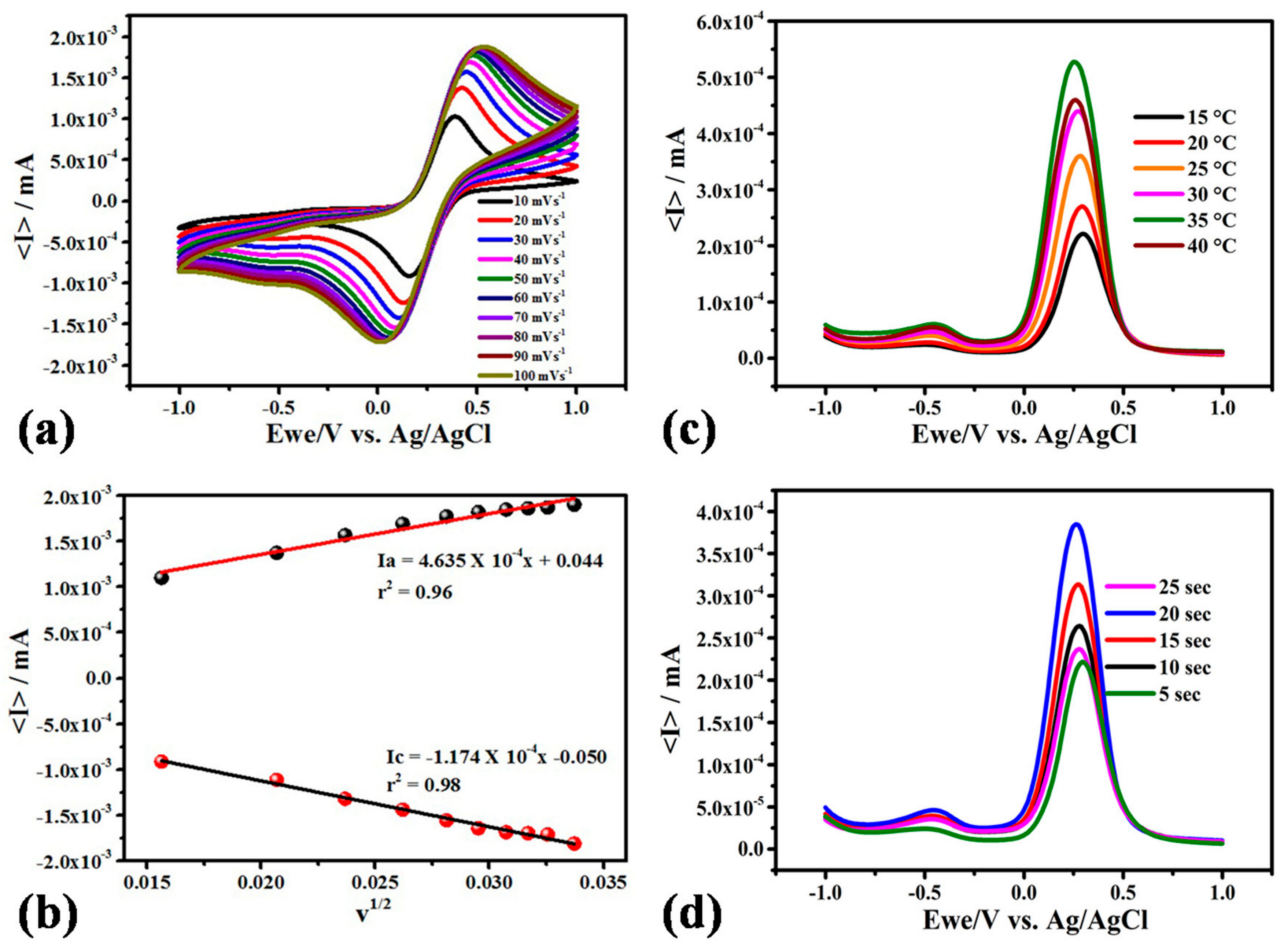
(**a**) Cyclic Voltammogram of probe modified electrode at different scan rates ranging from 10 to 100 mV/s. (**b**) Corresponding peak current vs square root potential ranging from 10 to 100 mVs^−1^. (**c**) Differential pulse voltammogram of probe modified electrode at different temperature ranging from 15 °C–40 °C. (**d**) Differential pulse voltammogram obtained at different response time ranging from 5–25 s. All optimization studies were done using 1 µM MB (pH 7.4) in 0.1 M KCl.

CV was recorded for ssDNA/MoSe_2_/FTO at the scanning rate of 10–100 mV/s. The increase in the anodic and cathodic peak current was directly proportional to the scan rate (Figure 4a). Figure 4b shows linear increase in peak current with the square root of scan rate and slope values; intercept and correlation coefficient are given in the equations below. The variation in the peak current (I) on the square root of the scan rate at 100 mV/s is expressed as:

Ia = 4.635 × 10^−4^x + 0.044 (r^2^ = 0.96) (1)

Ic = −1.174 × 10^−4^x − 0.050 (r^2^ = 0.98) (2)

Different temperature studies ranging from 15 to 40 °C were conducted as the temperature is an important parameter for the stability of DNA. As from the graph, it was depicted in the Figure 4c that there is an increase in the current signal upon an increase in temperature but after 35 °C there is a decrease in oxidation peak of MB. It was concluded that 35 °C is the optimum temperature for the sensor activity. The optimum temperature obtained made the sensor suitable to work in the room temperature. Different response time ranging from 5 to 25 s was chosen for the electrochemical study as shown in the Figure 4d. Increase in response was observed upon increasing the response time but there was a drastic decrease in current after 25 s which was due to that enough interaction of MB was already made with the DNA.

### 3.4. Analytical Performance of the DNA Biosensor

**Figure 5:**
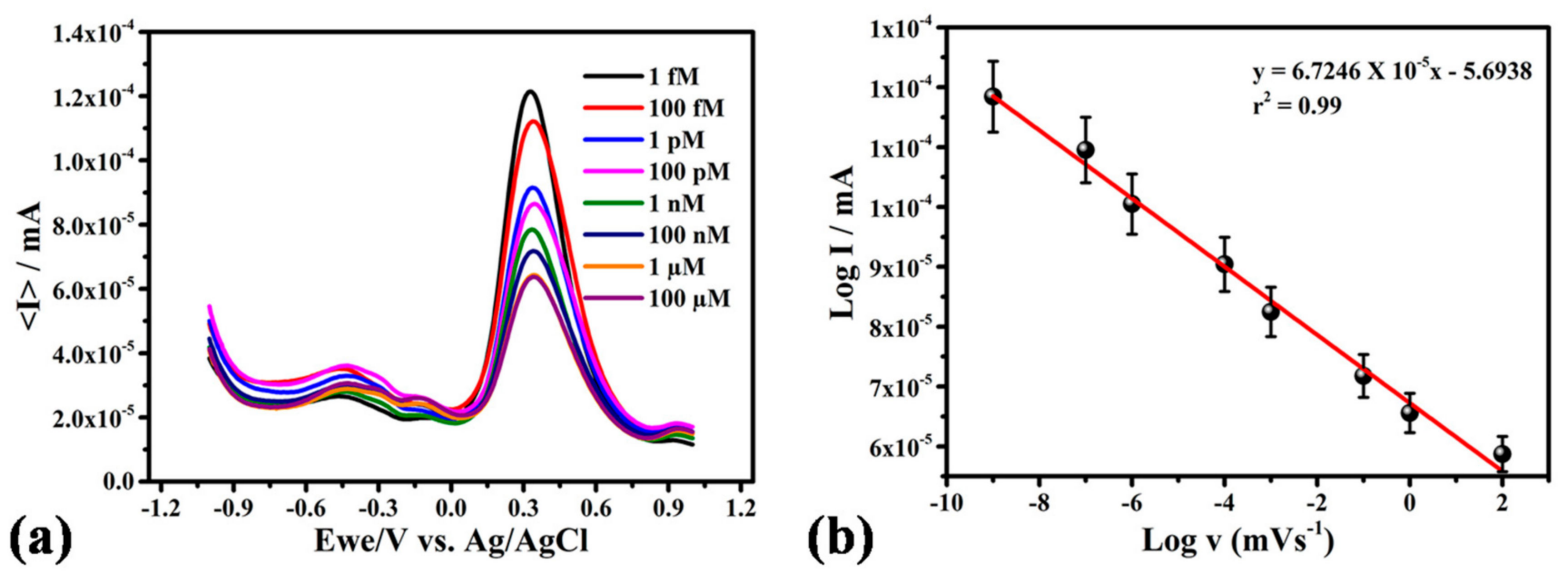
(**a**) Differential pulse voltammogram of different target DNA concentrations (*Escherichia coli* Shiga toxin) ranging from 1 fM–100 μM, at the scan rate of 100 mV/s. (**b**) Calibration plot obtained between log of the various concentrations of dsDNA and peak current.

DPV responses were recorded after subsequent additions of target DNA at different concentrations ranging from 1 fM to 100 mM in MB. It was depicted in Figure 5 that the anodic peak current of MB was decreased upon addition of target DNA which is ascribed to the more intercalation of MB in between the DNA bases which restricted its oxidation due to steric hindrances of bonded bases. The calibrations curve showed that there was a trend in the difference of current with the increase in target DNA concentration as R^2^ value is equivalent to the 0.99. There are only a few reported sensors for the detection of *Escherichia coli* Shiga toxin [26,27] and results proved that the MoSe_2_ significantly improved the sensitivity.

### 3.5. Selectivity and Stability of the DNA Biosensor 

**Figure 6:**
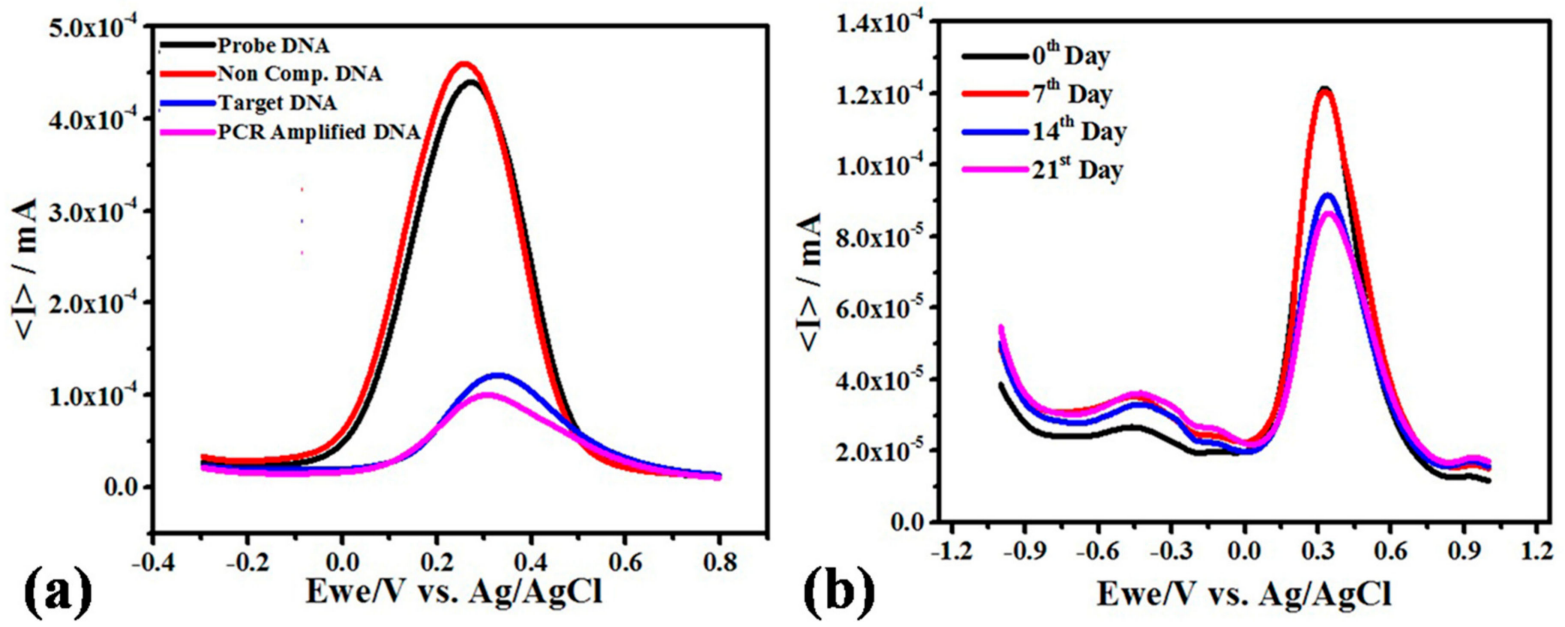
(**a**) Differential pulse voltammogram of ssDNA/MoSe_2_/FTO, ssDNA/MoSe_2_/FTO + non-complementary DNA, ssDNA/MoSe_2_/FTO + complementary DNA; (**b**) ssDNA/MoSe_2_/FTO performance for 5 measurements with four sets of electrodes at room temperature.

To investigate the selectivity of the biosensor, PCR denatured products of salmonella were employed as target DNA and non-complementary DNA. As shown in Figure 6a, the DPV response of the PCR products of salmonella was almost same as the commercial target DNA. While there was same current observed as that of the non-complementary DNA to the current response of probe DNA. This study concluded that the developed sensor is highly specific towards the target DNA of *Escherichia coli* Shiga toxin. The repeatability of the modified electrode was used to check the sensing response on the same day after repeating the experiment five times. All the conditions remained same i.e., DNA probe (20 µM) modified electrode, target concentration (1 fM), electrolyte (MB) and temperature (35 °C). Results obtained were found to be satisfactory i.e., 1.20 × 10^−4^ ± 1.06 × 10^−7^. Stability of the present sensor was checked by assessing same sets of five electrodes at 7th day, 14th day and 21st day (1 fM). The sensors showed in the Figure 6b was almost stable response up to the 7th day, but after 21st day the current was decreased by 33%. The sensing response checked at 21st day was found to be 6.39 × 10^−5^ ± 3.24 × 10^−7^.

### 3.6. Application of Geno-Sensor

**Figure 7:**
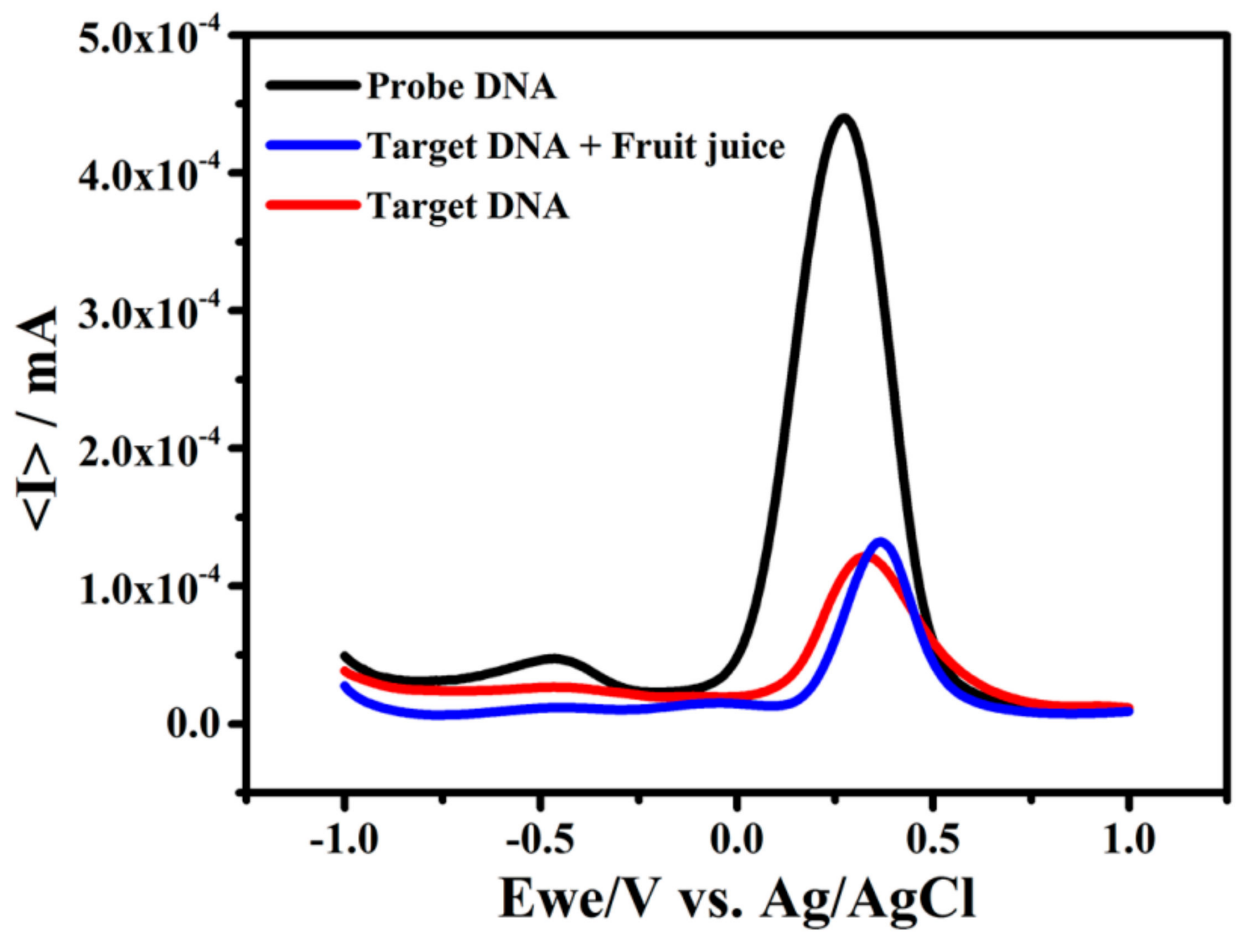
Differential pulse voltammogram of target DNA in real sample. All experiments were performed between −1.0 to +1.0 V at 100 mVs^−1^ in 1 µM MB (pH 7.4) containing 0.1 M KCl.

For checking the applicability and recovery of the sensor in real samples, fruit juices were spiked with the target DNA (1 fM) and DPV was performed. For recovery study, DNA (1 fM) was spiked in juice sample. The sensing response obtained was 1.31 × 10^−4^ mA and the current of 1 fM DNA without juice sample was found to be 1.21 × 10^−4^ mA. The obtained recovery was 92.36% indicating good accuracy of the proposed geno-sensor for the detection of *Escherichia coli* Shiga toxin DNA in real sample. It is also clearly depicted in Figure 7 that juice is showing insignificant interference which proved the applicability of the sensor in real samples.

## 4. Conclusions

Our study concluded that *Escherichia coli* Shiga toxin electrochemical DNA sensor was successfully fabricated using 2D nanomaterial MoSe_2_ nano-urchins/FTO. The developed geno-sensor exhibited high specificity and sensitivity towards *Escherichia coli* Shiga toxin and due to the conductive properties of MoSe_2_/FTO, the current response increased significantly upon addition of DNA specific to Shiga toxin thereby demonstrating a low detection limit of 1 fM for target DNA that shows the developed sensor is highly specific towards the target DNA. The present sensor shows insignificant interference when applied for the detection of target DNA in the real sample. The proposed FTO based geno-sensor can provide a promising platform for effective detection of *Escherichia coli* Shiga toxin.
